# Update and clinical management of anti-DNA auto-antibodies

**DOI:** 10.1515/almed-2021-0008

**Published:** 2021-04-26

**Authors:** Concepción González Rodríguez, MªBelén Aparicio Hernández, Inmaculada Alarcón Torres

**Affiliations:** Unidad de Bioquímica Clínica, Hospital Universitario Virgen Macarena Sevilla, Sevilla, Spain; Servicio Bioquímica Clínica y Análisis Cínicos, Complejo Asistencial Universitario Salamanca, Salamanca, Spain; Servicio Análisis Clínicos, Hospital Universitario Gran Canaria-HUGCDN, Gran Canaria, Spain

**Keywords:** anti-dsDNA, clinical biomarkers, systemic lupus erythematosus

## Abstract

Anti-deoxyribonucleic acid (DNA) antibodies in the clinical laboratory are intimately linked to the diagnosis and monitoring of systemic lupus erythematosus (SLE); however, the characteristics of the analytical methods and the properties of the antibodies themselves are heterogeneous. To review the definition and properties of anti-double-stranded anti-DNA (anti-dsDNA) antibodies, the adequacy of analytical methods, and the clinical requirements for this biomarker. Through PubMed we searched the existing literature with the terms anti-dsDNA, editorial, review, guideline, meta-analysis and SLE. The last search, anti-dsDNA and SLE restricted to the last two years. Information was expanded through related articles and those published in official state bodies related to anti-dsDNA and SLE. Clinical laboratory methods for anti-dsDNA analysis and their characteristics are analyze. The clinical utility of anti-dsDNA in its diagnostic, clinical association and follow-up aspects of SLE is reviewed. There is wide variability in analytical methods and deficits in standardization persist. They are part of the current SLE classification criteria and are used as markers in the follow-up of the disease. Their diagnostic usefulness improves when they are determined in antinuclear antibody (ANA)-positive patients. In follow-up, quantification is of interest, preferably with the same analytical method (given the deficits in standardization).

## Introduction

Systemic autoimmune rheumatic diseases have a low prevalence and are associated with a variety of signs and symptoms that make diagnosis challenging. Effective diagnostic biomarkers such as antibodies against anti-deoxyribonucleic acid have not yet been identified (DNA) [1].

Anti-dsDNA (anti-double-stranded DNA) antibodies have the ability to recognize all DNA structures present in chromatin, either relaxed or active. Thus, these antibodies recognize DNA, linear single-stranded DNA, double-stranded DNA (dsDNA) either circular or helical in its different forms. The most frequent forms, B dsDNA, with a right-handed double helix; Z dsDNA, with left-handed rotation; dsDNA, which is elongated with a long helix; and supercoiled helical dsDNA [2]. However, clinical associations have not been established for anti-dsDNA subpopulations targeted against the described forms of anti-dsDNA [2]. In this review, these forms will be referred to as anti-dsDNA. Anti-dsDNA is categorized into IgA, IgG, and Ig M; with IgG being the most clinically relevant and widely used antibody in clinical practice [3].

There is a wide spectrum of laboratory techniques available for the determination of anti-dsDNA, which challenges the use and interpretation of anti-dsDNA test results. The objective of this study is to review the definition and properties of anti-dsDNA antibodies, analyze the analytical and clinical properties of the measurement techniques available, and develop tools that facilitate the use of anti-dsDNA as a biomarker in systemic lupus erythematosus (SLE) patients.

## Materials and methods

A literature search of articles was performed on PubMed using the term anti-dsDNA and by type of publication, considering editorials, reviews, guidelines, or meta-analyses. A search of articles on anti-dsDNA and SLE restricted to the last two years was also carried out. A supplementary search for related articles and material on anti-dsDNA and SLE released by national entities was finally performed.

## Results

The information gathered is structured into five sections, namely: definition of anti-DNA antibodies and anti-dsDNA, properties, determination, clinical utility, and guidelines for their use.

### Definition

DNA is the main component of genetic material and stores genetic information. In eukaryotes and prokaryotes, DNA is compacted into a structure called nucleosome, which is composed of DNA and histones [4].

Anti-DNA antibodies recognize all DNA structures or components. However, in clinical terms and despite its limitations, the most relevant autoantibodies are anti-dsDNA [5].

SLE patients generate anti-dsDNA antibodies either when isolated or linked to proteins (e.g., histones) or integrated into more complex structures such as nucleosomes [4].

Antibodies against nucleosomes and histones have clinical relevance. Whereas anti-histone antibodies are associated with drug-induced lupus, the clinical role of anti-nucleosome antibodies is similar to that of anti-dsDNA antibodies and can be detected in the early stages of SLE, with a notably lower clinical implantation [6, 7].

### Properties

When a humoral response is initiated, the affinity of antibodies is low; however, as it progresses, the affinity of the antibody to its ligand (epitope) increases. In multi-functional molecules such as immunoglobulins, the strength with which immunoglobulin interacts with the antigenic molecule progressively increases via different ligands (avidity) [8]. The maturation and selection of immune response occurs in germinal centers, which manifests in the peptide forms of the variable region of immunoglobulins [9].

**Figure 1:**
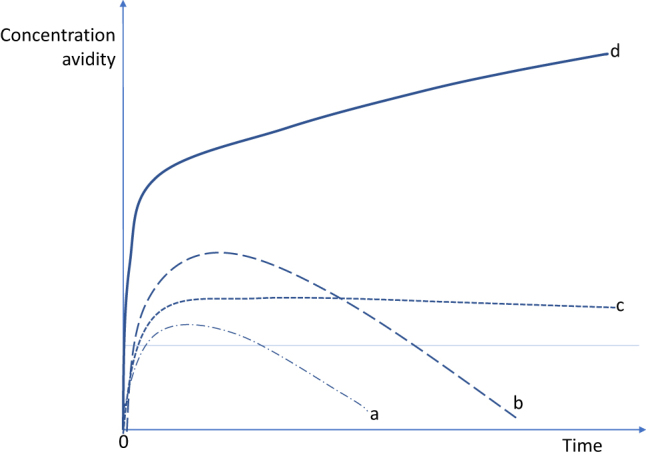
Theoretical anti-dsDNA response profiles. (a) A stimulus – an infection or autologous stimulus – triggers a transient response involving the production of low concentrations of low-avidity anti-dsDNA when the stimulus is short. (b) Transient response is stronger when the stimulus gets stronger. (c) Low but persistent anti-dsDNA concentrations with long-term exposure to the stimulus. (d) An immune response with sustained production of high concentrations of high-avidity anti-dsDNA antibodies in the adequate autoimmune context (modified by Rekvig [10]).

In a normal immunogenic context, a bacterial or viral infection can induce a transient immune response that recognizes the anti-dsDNA antibodies against the bacterium or virus and even of the host itself (Figure 1a). If the stimulus persists, anti-dsDNA antibodies remain present at low concentrations (Figure 1b). A very strong stimulus triggers a very strong transient response (Figure 1c). However, in the context of autoimmune diseases, the anti-dsDNA response recognizes anti-dsDNA antibodies, persists, and progresses. As a result, levels of anti-dsDNA antibodies increase, as do affinity and avidity. The latter is the most specific characteristic of SLE (Figure 1d) [10].

Concurrently to the infectious agent, other stimuli (exacerbated apoptosis, exposure to ultraviolet radiations, medications …) can generate anti-dsDNA antibodies in patients with SLE, cancer, or other autoimmune diseases [10], [11], [12]. There is evidence that anti-dsDNA antibodies recognize damaged DNA with a greater affinity, forming stronger immune complexes [13].

### Determination

#### Methods

In the past, anti-dsDNA antibodies were detected by enzyme-linked immunosorbent assay (ELISA) [14], Crithidia luciliae immunofluorescence test (CLIFT) [15], and radioimmunoassay [16]. However, new methods have been developed in the last decades such as fluorescent enzyme immunoassay (FEIA) [17], chemiluminescent enzyme immunoassays [18], and multiple-parameter assays (antibodies) (MPIA) [19, 20].

The most widely spread techniques in our environment are FEIA and CLIFT, followed by EIA, chemiluminescent immunoassay (CLIA), and MPIA, according to the methodology assessment reports of the quality program UK-NEQAS, with more than 600 participants [21]. The use of immunoblot or radioimmunoassay is very limited, as the first is not recommended [22] and the second has been replaced with non-radioisotope methods [23].

Farr’s technique, described in 1969 [24] is considered the gold standard. It is a quantitative, high-specificity technique for the detection of SLE. Its high specificity is credited to the precipitation of dsDNA/anti-dsDNA complexes with high salt concentrations (ammonium sulfate), by which high-avidity antibodies are selected [25]. This is the test with the best sensitivity/specificity balance for SLE. However, this technique involves the use of radioisotopes and detects IgA, IgG, and IgM anti-dsDNA but cannot differentiate them. A modified version of Farr’s method has been recently described, by which a fluorescent dye is injected into the dsDNA molecule (Farr-FIA). It has a good correlation with Farr’s radioisotope technique and reaches a diagnostic sensitivity and specificity of 53 and 100%, respectively [26].

In CLIFT, native dsDNA is strongly compacted in the kinetoplast. It has a high specificity and a high positive predictive value. However, its sensitivity is lower than that of other methods (especially in early SLE) [27].

In the ’80s, completely automated methods started to spread, first ELISA, then FEIA, MPIA, and CLIA. These methods have a higher sensitivity and lower specificity than CLIFT (it detects anti-dsDNA antibodies of lower affinity and avidity), with varying characteristics across manufacturers. High-salt concentrations such as wash buffer are used in ELISA [28] and CLIA [29], with the latter having a good sensitivity/specificity balance [18].

In the last years, a new flow cytometry-based method has been developed for detecting free anti-DNA autoantibodies and anti-DNA integrated into circulating immune complexes (endogenous DNA-anti dsDNA), with the latter having been integrated into routine screening and determination methods [30].

#### Characteristics

The characteristics of the most widely spread methods are shown in Table 1. The equipment most frequently used in the laboratories involved in the UK-NEQAS quality program are detailed next to each method, namely: Orgentec (EIA), Phadia 250 (FEIA), INOVA Quanta Flash (CLIA), and Luminex technology BioRad Bioplex 2200 (MPIA) [21].

**Table 1:** Characteristics of different anti-dsDNA tests (modified by Infantino et al. [33]).

Method	Antigen	Solid	Conjugate	Time for analysis	Detection	Calibration	Analytical range	Cut-offs
CLIA	Synthetic dsDNA	Para-magnetic beads	IgG	30 min	Quantitative	Curve	9.8–666.9 UI/mL	35–45 UI/mL ambiguous >45 IU/mL positive
MPIA	Synthetic dsDNA	Dyed magnetic beads	IgG	45 min	Quantitative	Curve	1–300 UI/mL	5–9 UI/mL doubtful ≥10 UI/mL positive
FEIA	Synthetic dsDNA	Micro-well	IgG	120 min	Quantitative	Curve	0.5–379 UI/mL	10–15 UI/mL doubtful >15 UI/mL positive
EIA	Synthetic dsDNA	Micro-well	IgG	120 min	Quantitative	Curve	0–200 UI/mL	20 UI/mL
IFI	Native dsDNA	Crithidea lucilliae	IgG	60 min	Qualitative	None	N/A	1/10

With respect to the substrate, CLIA [18, 31] and MPIA [19] use synthetic dsDNA bound to magnetic or paramagnetic particles. FEIA [17] and EIA [14] use synthetic or purified dsDNA bound to micro-wells. In CLIFT, dsDNA is exposed in the kinetoplast of the hemoflagellate Crithidia luciliae [15].

The conjugate generally allows for the detection of IgG antibodies. However, there are some exceptions, as some EIA analyzers detect IgG and IgM anti-dsDNA antibodies (Kallestad™) [32], whereas others detect IgA, IgG, and IgM anti-dsDNA (Orgentec) [33].

Table 1 shows other characteristics of the analytical methods most widely used in European clinical laboratories according to UK-NEQAS, with the data provided by reagent suppliers and Infantino et al. [33]. The characteristics of these methods are described below:

– The analysis takes longer with EIA and FEIA, as compared to MPIA and CLIA. When CLIFT is performed manually, the duration of the process is 60 min, although this time varies with automation and the time needed to read the sample by fluorescence microscopy.
– FEIA, MPIA, and CLIA methods enable continuous sample loading; whereas EIA and CLIFT work in lots.
– Calibration curves are stable during a variable period of time in FEIA, MPIA, and CLIA, whereas EIA requires a curve per lot, and no calibration curve is generated by CLIFT (a negative and positive control is performed for each lot).
– Detection is quantitative in all methods except for CLIFT, which is semi-quantitative with serial sample dilutions. Nevertheless, results can be expressed qualitatively by any of the methods used (positive/negative) by using a cut-off point established by each laboratory or manufacturer.
– The analytical range varies across manufacturers, with it being wider in CLIA methods.

Cut-offs and reference values are established by each laboratory. The table shows reference values provided by manufacturers. Although all methods are based on an international standard, there is persistent variability in cut-offs and reference intervals, which demonstrates a lack of standardization.

Comparative study of the analyzers used in the different methods analyzed demonstrates a moderate-to-high level of agreement among them, with kappa coefficients ranging from 0.47 and 0.68; clinical sensitivities for SLE between 5.7 (CLIFT) and 33.3% (EIA); specificities between 89.8 (MPIA) and 98.8% (CLIFT); positive likelihood ratio between 2.93 (MPIA) and 17.6 (CLIFT); and a non-significant negative likelihood ratio for any of the methods ranging between 0.71 (EIA) and 0.96 [33].

As expected, CLIFT showed a lower sensitivity by higher specificity and, when positive, indicates a higher likelihood of SLE (higher positive likelihood ratio) [34], [35].

Therefore, for the diagnosis of SLE, the Spanish Ministry of Health and Consumer Affairs recommends that anti-dsDNA antibodies are measured by CLIFT in a 1:10 dilution in patients with antinuclear antibodies [36]. CLIFT can be used as a second-line technique, as a confirmatory test, after a positive result has been obtained by an automated quantitative immunoassay. In this case, it is recommended that the two results are included in the laboratory report, even though they are inconsistent [37]. In general, blotting is not recommended for anti-dsDNA determination [22].

In addition, guidelines recommend that the method and reference values used for healthy controls and SLE patients are detailed in the laboratory report [3, 37].

In the presence of symptoms of SLE, a positive antinuclear antibody test, and an elevated anti-dsDNA titer, SLE is the first diagnostic option [36]. Once the diagnosis is established, lupus activity can be monitored using quantitative techniques. The quantitative results obtained must be reported. The same monitoring technique must be used during follow-up.

In active lupus nephritis, a recent study comparing Farr, ELISA, FEIA, and CLIA demonstrate a level of agreement of 95% between Farr and FEIA. With respect to lupus activity, Farr and FEIA showed a similar behavior [38].

#### Standardization

The first international standard for anti-dsDNA, Wo/80 was published by the World Health Organization (WHO) in 1985 [39]. This reagent of reference assigned International Units and improved the comparability of tests and laboratories. However, they are out of stock.

The standardization panel and the WHO have prepared and validated a new reference reagent, the so-called anti-dsDNA reference reagent for lupus (oligo-specific) 15/174. It has a nominal power of 100 units/ampoule, but it is not equivalent to the first international standard (Wo/80) and cannot be considered a continuation. It is available at NIBSC (https://www.nibsc.org/products/brm_product_catalogue/detail_page.aspx?catid=15/174) [40].

### Clinical utility

#### Diagnosis

As shown in Table 2, SLE classification criteria include the presence of anti-dsDNA antibodies. Thus, Table 2 provides a definition and the relative weight of anti-dsDNA antibodies in each criterion (1982 ACR classification criteria, revised 1997 ACR criteria, 2012 SLIIC criteria, and 2019 American College of Rheumatology–European League against Rheumatism (ACR–EULAR) classification criteria for SLE) [41], [42], [43], [44].

**Table 2:** Weight of anti-dsDNA antibodies in different criteria for systemic lupus erythematosus.

Criterion	Specifications	Relative weight
1982 ACR^a^	Abnormal native anti-ADN titer	1/11 criteria
1997 ACR^a^	Abnormal native anti-ADN titer	1/11 criteria
SLICC 2012^b^	Anti-dsDNA above laboratory reference values, except for ELISA: exceeding two times reference values.	1/17 criteria
2018 ACR–EULAR^b^	High-specificity anti-dsDNA	1/22 criteria

^a^SLE classification when: the patient meets four or more of the 11 criteria either simultaneously or serially for an indefinite observation period [41], [42]. “SLE classification” when: the patient meets four of the 17 criteria (11 clinical and six immunological) including at least one clinical and one immunological criterion either simultaneously or serially; or in the presence of histologically-confirmed nephritis consistent with SLE and positive antinuclear antibodies or anti-dsDNA [43]. ^b^SLE classification: in the presence of positive antinuclear antibodies at a titer of ≥1/80; if the total score is ≥10, the patient scores at least in a clinical domain and, in each domain, only the criterion with the highest weight is considered (Anti-dsDNA weight: 6 points) [44].

In SLICC 2012 criteria, anti-dsDNA antibodies were analyzed by immunoassay, CLIFT, and ELISA. The anti-dsDNA criterion was associated with an SLE with a sensitivity of 57.1% and a specificity of 95.9% in a sample of 716 patients with different autoimmune or inflammatory diseases [43]. In juvenile SLE (related to juvenile idiopathic arthritis), sensitivity was 52.2%, with a specificity of 100% [45].

In the 2019 ACR–EULAR criteria (Table 3), 22 criteria are categorized into different domains, with the variable weight assigned to each criterion. A patient will be diagnosed with SLE if they reach a score of 10 and exhibit a positive test for antinuclear antibodies at a titer ≥1/80 in HEp-2 cells or an equivalent test. Criteria and domains are shown in Table 1 and are not time-limited. They may have occurred before the progression of the disease (history of positive antinuclear antibody test). Criteria cannot be explained by conditions other than SLE, and at least a clinical domain must be scored. In the presence of more than one criterion in a clinical or immunologic domain, only the one with the highest weight will be considered. Anti-dsDNA antibodies are integrated into the domain of highly-specific antibodies. In their selection, the sensitivities and specificities of the 2012 SLICC (referred to above) and 1982 ACR classification criteria were considered (67 and 92%, respectively) [41], [46].

**Table 3:** 2019 ACR/EULAR criteria for systemic lupus erythematosus.

Clinical domains	Scores
**Constitutional domain**	
Fever	2
**Cutaneous domain**	
Non-cicatricial alopecia	2
Oral ulcers	2
Subacute cutaneous or discoid lupus	4
Acute cutaneous lupus	6
**Joint domain**	
Synovitis or pain in at least two joints and ≥30 min of joint stiffness	6
**Neurological domain**	
Delirium	2
Psychosis	3
Convulsions	5
**Serositis domain**	
Pleural or pericardial effusion	5
Acute pericarditis	6
**Neurological domain**	
Leukopenia	3
Thrombocytopenia	4
Auto-immune hemolysis	4
**Kidney domain**	
Proteinuria >0.5 g/24 h	4
Class II or V lupus nephritis	8
Class III or IV lupus nephritis	10

**Immunologic domains**	**Points**
**Antiphospholipid antibody domain**	
aCL or aβ2GP1 or lupus anticoagulant	2
**Complement protein domain**	
Low C3 or C4	3
Low C3 or C4	4
**Highly-specific antibodies**	
Anti-dsDNA	6
Anti-Sm	6

aCL, anti-cardiolipin; aβ2GP1, anti-β2 glycoprotein1; anti-dsDNA, anti-double-stranded DNA; anti-Sm, anti-Smith [41].

#### Association

Numerous studies have demonstrated that anti-dsDNA are pathogenic in lupus nephritis (LN) and are strongly associated with the disease [47]. Anti-dsDNA antibodies are involved in the pathogenesis of LN. They bind to the basal membrane and mesangial matrix in the form of circulating immune complexes composed of dsDNA and chromatin proteins [48]. However, circulating anti-dsDNA can also directly bind to kidney structures (fragments of chromatin exposed in the basal membrane or glomerular antigens with which anti-dsDNA cross-reacts). These two pathogenic mechanisms may co-exist and even predominate throughout the different stages of lupus nephritis [49, 50].

The clinical association with LN increases when anti-dsDNA antibodies co-exist with anti-nucleosomes and anti-histones, which contributes to differentiate SLE with LN from SLE without LN, which increases LN severity [51]. Other antibodies that can be associated with anti-dsDNA and NL are anti-fraction C1q of the complement and anti-Sm [39]. It has been postulated that positivity for these three types of antibodies with associated low C3/C4 concentrations and a reduced albumin/globulin ratio are suggestive and predictive of kidney involvement [52, 53]. The most recent studies show that high-morbidity anti-protein dsDNA-binding antibodies correlate with anti-dsDNA and lupus nephritis [53].

Some guidelines recommend LN response to treatment to be monitored by anti-dsDNA and complement test, urine sediment test, proteinuria in 24 h urine or protein/creatinine ratio, and serum creatinine [54]. However, other guidelines indicate that anti-dsDNA antibodies have low specificity for LN and have a limited clinical utility for follow-up of LN [36].

#### Follow-up

Anti-dsDNA and C3–C4 complement concentrations (C3/C4) are serological markers of SLE activity. Thus, to assess SLE activity, it is recommended that anti-dsDNA titers and levels of C3/C4 are both assessed [55]. Nevertheless, anti-dsDNA titration alone will not be predictive or confirmatory of SLE activity [3]. Determination intervals will be adjusted to the clinical status of the patient. If the disease is in clinical and analytical remission, 6–12-month follow-up is recommended based on the course of progression and intensity of the treatment. In clinically quiescent patients but with persistent analytical signs of activity, closer monitoring (3–4 months) is recommended [3, 36, 55].

Pregnancy is associated with alterations in C3/C4 concentrations. However, the determination of C3/C4 and anti-dsDNA concentrations during pregnancy is useful for monitoring lupus activity. This test must be performed upon suspicion of lupus activity [36]. Andreoli et al. indicate that elevated levels of anti-dsDNA concurrent to decreased levels of C3/C4 in pregnant patients are associated with an increased risk of an SLE flare-up (OR 5.3) and miscarriage [56].

Anti-dsDNA antibodies and C3/C4 are included in SLE activity indices. Table 4 shows one of the most widely used indices, SLEDAI-2K, which was developed by Gladman [57] and assesses the presence or absence of each activity descriptor in the previous 30 years. SRI-50, which is based on SLEDAI-2K, defines significant clinical improvement with respect to the basal status the same year when SLEDAI-2K descriptors decrease by ≥50% [58]. There are other SLE activity indices such as BILAG (*British Isles Lupus Assessment Group Index*) and BILAG 2004, ECLAM (*European Consensus Lupus Activity Measurement*), SLAM (*Systemic Lupus Activity Index*), SLAQ (*Systemic Lupus Activity Questionnaire*) that not always include anti-dsDNA elevation and hypocomplementemia among its descriptors [59]. In 2016, a panel of experts unanimously agreed that the presence of anti-dsDNA concurrent to hypocomplementemia is a marker of SLE activity (defined as above or below the laboratory reference value). As many as 93% of experts recommend that these markers should be added to the definition of SLE remission [60].

**Table 4:** Systemic lupus erythematosus activity index (SLEDAI 2000) or SLEDAI-2K.

Descriptor	Score	Descriptor	Score
Convulsions	8	Proteinuria^c^	4
Psychosis	8	Piuria^d^	4
Organic brain syndrome	8	Rash	2
Visual alterations	8	Allopecia	2
Cranial nerve alterations	8	Mucosal ulcers	2
Lupus headache	8	Pleuritis	2
Stroke	8	Pericarditis	2
Vasculitis	8	Low complement^e^	2
Arthritis	4	High anti-dsDNA^f^	2
Myositis	4	Fever	1
Urinary casts^a^	4	Leukopenia^g^	1
Hematuria^b^	4	Thrombocytopenia^h^	1

^a^Granular or hematic cylinders; ^b^More than five RBCs/field; ^c^More than 0.5 g proteins/24 h; ^d^More than Leukocytes/field; ^e^Complement CH50, C3 or C4; ^f^Anti-dsDNA exceeding the reference range; ^g^Leukocytes <3 × 10^9^/L; ^h^Platelets <100 × 10^9^/L. Onset, recent or persistent SLE is determined based on the descriptor.

At present, efforts are focused on establishing a definition to differentiate reduced SLE activity from remission. Complete remission is established when SLE is inactive without medication or only with anti-malaria drugs such as hydroxychloroquine. For the moment, a definition of reduced activity has not yet been established, and some definitions consider SLEDAI-2K and the drugs needed to maintain reduced SLE activity. Definitions have been provided for minimal SLE activity (MDA), low SLE activity (LDA), and low SLE activity status (LLDAS). Serological activity (measured based on anti-dsDNA and C3/C4) is included in LLDAS. In contrast, the definition of MDA and LDA only considers clinical variables [61].

Recently, the Mexican Association of Rheumatology recommends that low complement levels, elevated levels of anti-dsDNA, and slightly increased C-reactive protein are considered to determine whether the presence of fever is associated or not with SLE activity in a patient [54].

A variety of indices are available to assess response to treatment [58]. However, the role of anti-dsDNA in treatment response assessment is controversial. Experiences are inconsistent because of the heterogeneity of measurement equipment, among other factors. Some experts advocate for the use of anti-dsDNA antibodies alone or in combination with other clinical or analytical parameters to monitor patient response to treatment (biological or nonbiological) [1, 62, 63].

### Guidelines for use

In this last section, the authors provide guidelines for an adequate use of anti-dsDNA based on available evidence.

The clinical and pathogenic interest of anti-dsDNA increases when anti-dsDNA of high affinity and avidity are detected. In general, high-avidity and high-affinity antibodies are detected with Farr (unused) and CLIFT, which have a high specificity. Therefore, the use of CLIFT is recommended for the diagnosis of SLE. Nevertheless, this technique can be used as a second-line technique after a positive quantitative test. If CLIFT cannot be used, a test result exceeding two times the upper limit of the reference value for the control group will be considered positive. The clinician should be familiarized with the technique and laboratory reference values.

For the diagnosis and upon clinical suspicion of SLE, anti-dsDNA should be determined in patients with positive antinuclear antibodies, and results for the two types of antibodies should be reported. Exceptionally, on high suspicion of SLE, anti-dsDNA can be directly measured because anti-dsDNA can be detected in patients negative for antinuclear antibodies based on the technique and cut-off used, as fixation methods and the characteristics of analytical methods may vary. For example, in HEp-2 cells, dsDNA is found in nuclei bound to histones and other nuclear proteins and fixed to the glass plate using organic solvents. In contrast, in specific methods, native purified or recombining anti-dsDNA is bound to a solid plastic plate without using any substance.

Anti-dsDNA antibodies only have diagnostic value in SLE and test results should always be interpreted in relation to other clinical data. Anti-dsDNA antibodies have high specificity and are included in immunological classification criteria. An elevated concentration of anti-dsDNA in people with clinical signs of SLE and a positive antibody test is suggestive of SLE as the first diagnostic option. However, a negative result does not exclude SLE, as it has a low sensitivity and negative likelihood ratio.

Quantitative anti-dsDNA determination is recommended – always using the same method – for monitoring SLE patients.

Anti-dsDNA is associated with lupus activity, especially when detected at high concentrations using high-specificity methods and are concurrent to other clinical or analytical signs of SLE activity. Elevated levels of anti-dsDNA alone are not predictive of SLE activity.

Anti-dsDNA and complement C3/C4 levels are included in SLE activity indices and should be both determined on suspicion of SLE activity, also in pregnant SLE patients.

Anti-dsDNA is correlated with LN and has a relative utility for diagnosis and assessment of SLE activity. As it occurs with SLE activity, high concentrations and specificity improve the association and clinical utility of anti-dsDNA in LN. However, test results should be interpreted in relation to other kidney disease markers.

## Conclusions

There is a wide range of analytical methods for the determination of anti-dsDNA antibodies but a lack of standardization. Not all methods detect antibodies with the same level of avidity and affinity. Anti-dsDNA with high avidity and affinity in antinuclear antibody (ANA)-positive patients have a high diagnostic specificity for SLE. Anti-dsDNA determination – always using the same technique – is useful for follow-up of SLE (because of the lack of standardization). Finally, variability and progress in the measurement methods used modify their clinical characteristics.
